# Novel insights into vancomycin-loaded calcium sulfate and negative pressure wound therapy in preventing infections in open fractures

**DOI:** 10.1186/s13018-024-04931-5

**Published:** 2024-08-29

**Authors:** Bei Jia, Rui Xue, Jia Li, Jichao Guo, Jianning Liu

**Affiliations:** 1 Nosocomial Infection Management Department, Hebei Medical University First Hospital, No. 89 Donggang Road, Yuhua District, Shijiazhuang City, China; 2 Department of Orthopaedic Suigery, Hebei Medical University Third Hospital, No. 139 ZiQiang Road, QiaoXi District, Shijiazhuang City, Hebei Province China

**Keywords:** Open fractures, Staphylococcus aureus infections, Vancomycin-Loaded Calcium Sulfate, Negative pressure wound therapy, Macrophage polarization, M2

## Abstract

**Background:**

Open fractures are challenging due to susceptibility to Staphylococcus aureus infections. This study examines the impact of Vancomycin-Loaded Calcium Sulfate (VLCS) and negative pressure wound therapy (NPWT) on macrophage behavior in enhancing healing and infection resistance. Both VLCS and NPWT were evaluated individually and in combination to determine their effects on macrophage polarization and infection resistance in open fractures.

**Methods:**

Through single-cell RNA sequencing, genomic expressions in macrophages from open fracture patients treated with VLCS and NPWT were compared to a control group. The analysis focused on MBD2 gene changes related to macrophage polarization.

**Results:**

Remarkable modifications in MBD2 expression in the treatment group indicate a shift towards M2 macrophage polarization. Additionally, the combined treatment group exhibited greater improvements in infection resistance and healing compared to the individual treatments. This shift suggests a healing-promoting atmosphere with improved infection resilience.

**Conclusions:**

VLCS and NPWT demonstrate the ability to alter macrophage behavior toward M2 polarization, which is crucial for infection prevention in open fractures. The synergistic effect of their combined use shows even greater promise in enhancing outcomes in orthopedic trauma care.

**Supplementary Information:**

The online version contains supplementary material available at 10.1186/s13018-024-04931-5.

## Background

Open fractures are severe injuries caused by violent external forces that result in skin rupture and direct communication between the fractured ends of the bone and the external environment [1]. Due to the exposed wound, bacteria easily infiltrate, leading to infections and serious complications such as osteomyelitis [2]. In particular, Staphylococcus aureus is one of the main pathogens responsible for fracture infections [3]. Open fracture infection has become a pressing challenge in clinical practice, as it not only seriously threatens the patient’s life but also increases the burden on healthcare [4]. Therefore, it is of vital clinical significance to propose effective prevention and treatment strategies.

Vancomycin-Loaded Calcium Sulfate is a broad-spectrum antibiotic that inhibits bacterial cell wall synthesis, suppressing bacterial growth and exhibiting strong inhibitory effects against various bacteria, including Staphylococcus aureus [5]. Previous studies have confirmed the significant effectiveness of Vancomycin-Loaded Calcium Sulfate in treating open fracture infections [6]. However, we have limited knowledge about how Vancomycin-Loaded Calcium Sulfate regulates the immune response of the body, particularly its specific mechanism of affecting macrophage polarization to resist infections.

Macrophages, as important components of the immune system, can be categorized into two types: M1 and M2 [7]. M1 macrophages exhibit strong antibacterial and inflammatory responses, while M2 macrophages primarily participate in the resolution of inflammation and tissue repair [8]. In recent years, there has been increasing attention to the role of macrophage polarization in infection control [9]. Methyl-CpG-binding domain protein 2 (MBD2) is an important transcriptional regulator that has been shown to play a crucial role in macrophage polarization [10]. However, the exact mechanism of utilizing MBD2 to mediate macrophage polarization and combat open fracture infections remains unresolved [11].

Negative pressure drainage is a novel medical device that promotes wound healing by generating negative pressure at the wound site [12]. Recent studies have shown that negative pressure drainage not only clears the source of infection within the wound but also enhances the body’s immune response to increase its ability to fight infections [13]. However, the interaction and synergistic effects of negative pressure drainage with Vancomycin-Loaded Calcium Sulfate in combating open fracture infections, particularly their impact on macrophage polarization, remain unresolved.

In summary, through the use of single-cell transcriptome sequencing technology, we systematically investigated how Vancomycin-Loaded Calcium Sulfate regulates macrophage polarization mediated by methyl-CpG-binding domain protein 2 to combat open fracture infections and explored the role of negative pressure drainage. We hope that this study will provide new theoretical foundations and therapeutic targets for the prevention and treatment of open fracture infections, ultimately delivering tangible treatment outcomes for patients.

## Materials and methods

### Ethics statement

All clinical samples were collected with the approval of the relevant Hebei Medical University Third Hospital ‘s ethics committee, and patients provided informed consent. Animal experiments adhered to the “Ethical Regulations for Animal Experiments” and followed the guidelines of Hebei Medical University Third Hospital’s animal experiment committee, which granted approval for all experimental procedures. We are committed to minimizing animal suffering during the experiments, and all animals were handled humanely following the conclusion of the experiments.

### Macrophage culturing and treatment

THP-1 cells (1 × 10^6^) (CBP60518, Nanjing Ke Bai Biotechnology Co., Ltd.) were seeded into a 6-well plate and treated with 150 nM PMA (P8139, Sigma-Aldrich, USA) for 24 h to obtain M0 macrophages. M0 macrophages were differentiated into M1 macrophages by culturing them for an additional 24 h in RPMI-1640 medium (11,875,176, Gibco) containing 10 pg/mL LPS (L5293, Sigma-Aldrich) and 20 ng/mL IFN-γ (SRP3058, Sigma-Aldrich). M0 macrophages were differentiated into M2 macrophages by culturing them for an additional 24 h in RPMI-1640 medium (11,875,176, Gibco) containing 20 ng/mL IL-4 (SRP4137, Sigma-Aldrich) and 20 ng/mL IL-13 (SRP3274, Sigma-Aldrich). The cells were cultured in a CO2-enriched incubator (DHP-9162, Jiecheng Experimental Instrument, Shanghai, China) at 37 °C with 5% CO2 and saturated humidity. The culture medium was replaced with fresh medium every 1–2 days, and passaging was carried out when cell confluence reached 80-90% [14].

Van-CaSO4 (VS-1010, Biomatlante) was sterilized by UV irradiation and then soaked in MEMα medium (32,571,101, Gibco) supplemented with 10% FBS at 37 °C in a sealed incubator for 72 h. The obtained leachate was filtered and stored at 4 °C for later use. The Van-CaSO4 solution obtained was directly added to the macrophages, designated as the Van-CaSO4 group, while the macrophages treated with medium without Van-CaSO4 were used as the control group [15].

### Flow Cytometry-based cell sorting and single-cell transcriptome sequencing

We employed the BD FACSAria III flow cytometer to perform single-cell sorting of macrophages from the control and treatment groups, ensuring that each cell was sequenced independently [16]. Initially, the sorting parameters of the flow cytometer were adjusted, followed by the preparation of single-cell suspensions from the samples to be sorted. Sorting was then performed, with individual cells being expelled through the flow cytometer by adjusting the parameters accordingly. For single-cell RNA sequencing of macrophages from the control and treatment groups, we utilized the Chromium Single Cell 3’ Reagent Kits v3 from 10X Genomics. The sequencing depth was set at 10,000 cells. The raw sequencing data generated by the sequencer were processed using the standard workflow of cell range, aligning the obtained sequences to the human reference genome (GRCh38) [17]. Please refer to Figure S1 for the bioinformatics processing of the sequencing data.

### Dimensionality reduction and clustering analysis

The R package Seurat v3 was utilized for the standardization, feature selection, dimensionality reduction (e.g., PCA and UMAP), clustering, and differential expression analysis of single-cell data [18]. The data of the top 2000 highly variable genes were scaled using the FindVariableFeatures function in Seurat to identify 12 principal components of utmost importance. FindNeighbors in Seurat were employed to calculate the distances between cells and determine their clustering, with FindCluster utilized to obtain cell subtypes. Furthermore, the UMAP algorithm enabled unsupervised clustering and unbiased visualization of cell subpopulations. Through this analysis, we aimed to explore the heterogeneity within the single-cell population, identify distinct cell subtypes, and analyze their expression characteristics [19].

### Differential gene expression analysis

Differential gene expression between different subgroups was analyzed using the FindAllMarkers function in Seurat. The non-parametric Wilcoxon rank-sum test was employed to obtain *p*-values for comparisons, and all gene-adjusted *p*-values were corrected using Bonferroni correction. Differential genes with |LogFC|>1 and P adjusted < 0.05 were selected after logarithmic transformation and scaling, and their expression patterns were visualized using a heatmap [20].

### Functional enrichment analysis

Functional enrichment analysis of Gene Ontology (GO) and Kyoto Encyclopedia of Genes and Genomes (KEGG) was performed using the R package clusterProfiler. Differential expression gene lists were inputted to identify the enrichment of these genes in terms of biological processes (BP), cellular components (CC), molecular functions (MF), and KEGG pathways. The results display enriched GO or KEGG terms, along with their corresponding *p*-values and the number of enriched genes [20].

### Exploring the differentiation trajectory of macrophages through network analysis

We utilized differential gene expression data to investigate the interactions between proteins encoded by genes. In order to do so, we accessed the String database (https://cn.string-db.org/) and identified the protein-protein interactions. Additionally, we employed the R software to identify hub genes within the obtained protein interaction network, as these genes are likely to play a critical role in regulating macrophage polarization and infection response [21]. Furthermore, the monocle package in R was used to study the temporal trajectory of macrophage differentiation.

### Gene regulatory networks and hub TF

In this study, we employed the SCENIC method to identify key transcription factors (TFs) in the gene regulatory network (GRN). The process involved two steps. Firstly, we used SCENIC to predict the GRN and TFs, which consisted of three main stages: construction of co-expression modules, motif analysis for direct relationships identification, and calculation of RAS (regulon activity score) using AUCell. The corresponding GRN and TFs were identified from macrophages. In the second step, hub TFs and their target genes in the disrupted GRN were determined using WGCNA. Finally, we performed a cross and combined analysis of TF-target gene pairs. TFs that were identified in the intersection analysis were referred to as central TFs, and those in the union analysis were designated as key TFs. These TFs were further investigated for subsequent analysis [22].

### Gene knockout or overexpression

THP-1 cell groups were divided into the following: MBD2-WT group (untreated), MBD2-KO-1 group (MBD2-1 gene knocked out using CRISPR-Cas9 system), MBD2-KO-2 group (MBD2-2 gene knocked out using CRISPR-Cas9 system), MBD2-KO-3 group (MBD2-2 gene knocked out using CRISPR-Cas9 system), oe-NC group (control group infected with lentivirus), and oe-MBD2 group (MBD2 overexpression group infected with lentivirus).

gRNAs for MBD2 were designed (Table 1) and CRISPR-Cas9 system was prepared using LentiCRISPRv2 vector (Addgene, #52,961) or pLV overexpression plasmid containing MBD2 (GeneCopoeia, EX-Z5714-Lv105). The appropriate lentivirus carrying CRISPR-Cas9 system or overexpression plasmid with MBD2 (MOI = 10, working titer approximately 5 × 10^6^ TU/mL) was introduced into macrophages using the lentivirus packaging system (Sigma-Aldrich, SHC001). Stable transfected cell lines were selected using 3 µg/ml Puromycin (540,222, Sigma-Aldrich), and gene knockout or overexpression efficiency was validated using western blot or qPCR (TaqMan Gene Expression Assays, Thermo Fisher Scientific).

**Table 1:** gRNA sequence for MBD2

Name	Sequence (5’-3’)
gMBD2-1	TCGCCCCGTCCCCGGTGAG
gMBD2-2	GCCGGTCCCTTTCCCGTCG
gMBD2-3	CAAGGCCGCTGCCGCCACT

### Flow cytometry

Flow cytometry was used to assess the proportion of M2 macrophages. THP-1 cells were collected in cold PBS. The cells were then resuspended in a flow cytometry buffer (1x PBS with 1% BSA) and stained with anti-CD11b antibody (biolgend, #982,614, 1:100) and anti-CD206 antibody (biolgend, #321,132, 1:100) on ice for 30 min. Flow cytometric analysis was performed using a C500 instrument (Beckman, USA). The data were analyzed using FlowJo software [23].

Mouse fracture tissue was collected in cold PBS and processed as previously described. Briefly, the samples were mechanically sliced using a McIlwain tissue chopper (Mickle Laboratory Engineering). Then, the tissue was enzymatically digested for 1 h at 37 °C in a shaking water bath using 3 mg/ml collagenase type A (10,103,578,001, Roche) and 25 µg/ml DNase I (11,284,932,001, Roche) in serum-free culture medium. The cells were then resuspended in the flow cytometry buffer (1x PBS with 1% BSA) and stained with anti-CD11b antibody (biolgend, #101,206, 1:100) and anti-CD206 antibody (biolgend, #141,706, 1:100) on ice for 30 min. Flow cytometric analysis was performed using a C500 instrument (Beckman, USA). The data were analyzed using FlowJo software [23].

### Establishment of an animal model for open fracture infection

We utilized 12-week-old adult male C57BL/6 mice (WS020004, Zhonghong Boyuan) to create an animal model for open fracture infection. The care and use of the animals followed the approved protocols of our institution’s animal experimental committee (approval number: Z2023-018-1). After administering pentobarbital sodium to induce general anesthesia in the mice, we performed surgery to create an open fracture model in the femur.

Construction of the Mouse Fracture Infection Model (MRSA): The skin of the right leg of the mice was depilated and disinfected with alcohol. An incision was made on the right tibia, and the patella was laterally displaced. Using a 25-gauge needle, a hole was pre-drilled on the cortical bone of the proximal tibia. A transverse, non-crushing mid-diaphyseal fracture was then created on the distal end of the tibia using a surgical scalpel. A 0.35 mm needle was inserted in the proximal tibia to stabilize the fracture, with efforts made to minimize bleeding and surrounding tissue damage. A 5 µl suspension of 1 × 10^8^ CFUs of Staphylococcus aureus in PBS was directly inoculated at the site of the fracture [24]. Excess moisture at the fracture site was removed using sterile gauze, followed by wound closure. The sterile fracture group underwent only the fracture procedure without Staphylococcus aureus infection.

The successfully modeled Staphylococcus aureus infected mice were randomly divided into 4 groups, with 6 mice in each group: MRSA group (mice with the fracture infection model without any treatment), MRSA + Van-CaSO4 (mice with the fracture infection model and Vancomycin-Loaded Calcium Sulfate treatment), MRSA + NPWT group (mice with the fracture infection model and negative pressure wound therapy treatment), and MRSA + Van-CaSO4 + NPWT group (mice with the fracture infection model treated with Vancomycin-Loaded Calcium Sulfate and negative pressure wound therapy). The negative pressure wound therapy group received treatment at the fracture site using a negative pressure drainage device (M8275058/5, KCI V.A.C. Therapy System, www.kci-world.org/). The Vancomycin-Loaded Calcium Sulfate group had Vancomycin-Loaded Calcium Sulfate implanted at the fracture site (VS-1010, Biomatlante) and underwent X-ray imaging in the fourth week of treatment using the Kodak in vivo Multispectral Imaging System FX Pro to assess bone repair.

At each time point, bacterial colony-forming units (CFUs) were determined using an in vitro bacterial culture method. Briefly, tissues were vortexed in 1 ml of 0.3% Tween-80 in Tryptic Soy Broth (TSB) for 5 min, followed by a 5-minute incubation and then collection of the supernatant for CFU counting after overnight incubation on culture plates. At the end of the experiment, mice were euthanized under anesthesia, and the femurs were collected for subsequent histological analysis to evaluate bone repair and infection status.

### ELISA detection

On the 5th day post-infection, blood serum was collected from mice and centrifuged at 3000 rpm, 4 °C for 15 min to obtain the supernatant. The Pierce™ BCA Protein Assay Kit (Catalog Number: 23,225, Thermo Fisher Scientific, USA) was used to quantify total protein concentration. After 48 h, the supernatant from the culture was collected, and the expression levels of corresponding proteins were determined according to the instructions provided in the ELISA kits for G-CSF (ab197743, Abcam, Cambridge, UK), IL-6 (ab222503, Abcam, Cambridge, UK), IL-12 (p40) (ab236717, Abcam, Cambridge, UK), and IL-12 (p70) (ab119531, Abcam, Cambridge, UK) [24].

### X-ray detection of mouse bone tissue

To ensure the accurate placement of implants, lateral X-ray images of the femur were obtained using a high-resolution X-ray system (Faxitron LX-60 DC-12 imaging system). The images were also captured on the 28th day after the surgery. A trained orthopedic surgeon assessed the high-resolution X-ray images to determine whether the vancomycin-coated implants had any impact on bone structure and implant stability after 4 weeks. The evaluator was blinded to the treatment group [25].

### RT-qPCR

Total RNA from each group of cells was extracted using TRIzol (ThermoFisher, USA) (Catalog number 15,596,026), and the concentration and purity of the extracted RNA were assessed using the NanoDrop2000 spectrophotometer (ND-2000, ThermoFisher, USA). Subsequently, cDNA was synthesized from the mRNA using the PrimeScript RT reagent Kit (Takara, Japan) (Catalog number RR047A), following the manufacturer’s instructions. For the synthesized cDNA samples, RT-qPCR analysis was performed using the Fast SYBR Green PCR Master Mix (ThermoFisher, USA) (Catalog number 11,736,059), with three replicates for each sample. The housekeeping gene GAPDH was used as the reference gene for mRNA, and the fold change in gene expression between the experimental and control groups was calculated using the 2^−ΔΔCt^ method, where ΔΔCT = ΔCt experimental group - ΔCt control group, and ΔCt = Ct target gene - Ct reference gene. In this case, Ct refers to the number of amplification cycles required for the real-time fluorescence intensity to reach the predetermined threshold, at which point amplification follows a logarithmic growth pattern [26]. The experiment was repeated three times. The primer sequences can be found in Table 2.

**Table 2:** RT-qPCR primer sequences

Gene	Primer
MBD2 (human)	F 5’- GAATGAACAGCCACGTCAGC-3’
	R 5’-GGTTCTTTTCCACAGCAGCG-3’
Nos2 (human)	F 5’-GCCATAGAGATGGCCTGTCC-3’
	R 5’- TGCATCCAGCTTGACCAGAG-3’
Cd86 (human)	F 5’- CTTCCTGCTCTCTGCTAACTTC-3’
	R 5’- GCTGATGGAAACGTCGTACA-3’
Arg1 (human)	F 5’- ACTTAAAGAACAAGAGTGTGATGTG-3’
	R 5’- CATGGCCAGAGATGCTTCCA-3’
Cd206 (human)	F 5’- GCCTCGTTGTTTTGCGTCTT-3’
	R 5’- GAGAACAGCACCCGGAATGA-3’
GAPDH (human)	F 5’-GAGAAGGCTGGGGCTCATTT-3’
	R 5’-AGTGATGGCATGGACTGTGG-3’

Note: F, forward; R, reverse

### Western blot

Cells or tissues were lysed in RIPA lysis buffer (P0013B, Beyotime Biotechnology) to extract total protein. The lysates were incubated on ice for 30 min and then centrifuged at 8000 g, 4℃ for 10 min. The supernatant was collected. The protein concentration was determined using the BCA assay kit (A53226, ThermoFisher, USA). The proteins were separated by polyacrylamide gel electrophoresis and transferred to a PVDF membrane (IPVH85R, Millipore, Germany) by wet-transfer method. The membrane was blocked with 5% BSA at room temperature for 1 h and incubated overnight at 4℃ with primary antibodies including rabbit anti-MBD2 (ab188474, 1:1000, Abcam), Nos2 (ab178945, 1:1000, Abcam), Cd86 (ab112490, 1:1000, Abcam), Arg1 (ab203490, 1:1000, Abcam), Cd206 (ab64693, 1:1000, Abcam), and rabbit anti-GAPDH (ab9485). Following three washes with TBST (each for 10 min), the membrane was incubated with HRP-conjugated goat anti-rabbit IgG H&L secondary antibody (ab97051, 1:2000, Abcam, Cambridge, UK) for 1 h. After washing with TBST, the membrane was placed on a clean glass plate. Equal volumes of solution A and solution B from the ECL fluorescent detection kit (abs920, Aibobio, Shanghai, China) were mixed and then dripped onto the membrane in a dark room. The membrane was analyzed using Quantity One V4.6.2 software from Bio-Rad (USA) to determine the relative protein content, represented as the grayscale intensity of the corresponding protein bands normalized to the intensity of the GAPDH protein band [26]. The experiment was performed three times, and the mean values were calculated.

### H&E staining

The tissue samples were sequentially immersed in 70%, 80%, 95%, and 100% alcohol solutions to remove moisture. To achieve transparency, the samples were subjected to histoclear treatment (National Diagnostics, HS-200). After being immersed in the solution for 10 min, the samples were rinsed with water and subsequently soaked in a solution containing hematoxylin for 10 min (Sigma-Aldrich, H9627). They were then rinsed with water again and immersed in a solution containing eosin for 1–2 min (Sigma-Aldrich, product number: E4382). After another rinse with water, the samples were mounted using the CV5030 coverslipped from Leica Biosystems, followed by observation under the Axio Imager.A2 optical microscope from Zeiss [27].

### Safranin O staining

The tissues were fixed in 10% neutral buffered formalin and decalcified in a 0.5 M ethylenediaminetetraacetic acid (EDTA) solution, pH 8.0 (American Bio, Natick, MA, USA). The tissues were then dehydrated using graded alcohols, cleared with xylene, and processed in paraffin using the Tissue Tek VIP tissue processor (Sakura Finetek, Torrance, CA, USA). Tissue sections of 5 μm thickness were cut and mounted on glass slides, followed by staining with safranin O dye (350-M, Sigma-Aldrich). Histological images were captured using a Zeiss Axio Imager.A2 optical microscope [28].

### Statistical analysis

The statistical analysis in this study was performed using the R software. Continuous variables were presented as mean ± standard deviation, while categorical variables were presented as frequencies and percentages. To compare differences between the two groups, either the Student’s t-test or the Mann-Whitney U test was employed, depending on the adherence of the data to the assumptions of normal distribution and homogeneity of variances. For comparisons involving three or more groups, either a one-way analysis of variance (ANOVA) or the Kruskal-Wallis H test was used. If the analysis of variance yielded significant results, Tukey’s Honestly Significant Difference post hoc test was subsequently conducted to determine specific pairwise differences. Processing and analysis of single-cell transcriptomic data were conducted using the Seurat package. Cell clustering was performed using PCA and UMAP. The identification of differentially expressed genes was accomplished using the Wilcoxon rank sum test. Throughout all tests, *p*-values less than 0.05 were considered statistically significant.

## Results

### Identification and annotation of macrophage subpopulations in response to vancomycin-loaded calcium sulfate treatment

We first isolated macrophages from the Vancomycin-Loaded Calcium Sulfate treatment group and the control group and performed single-cell transcriptome sequencing on both groups. The raw data was analyzed using the Cellranger software to obtain barcode, gene, and cell information. The mitochondrial quality control (QC) metrics were calculated using the PercentageFeatureSet function, where we used all genes in the MT set as mitochondrial genes. We filtered out cells with more than 2000 or fewer than 200 genes, as well as cells with a mitochondrial gene proportion greater than 5% (Figure S2A, B).

After sequencing depth and mitochondrial gene expression normalization, we filtered out 2000 highly variable genes for further analysis. Since most of the cells had similar gene expression, we aimed to identify the highly variable genes that represented the main differences in the data (Figure S2C). Following feature selection, we applied principal component analysis (PCA), a dimensionality reduction algorithm, to compress the expression matrix of the highly variable genes (Figure S2D). We also used the ElbowPlot to sort the standard deviations of the principal components (PCs) (Figure S2E).

**Fig. 1:**
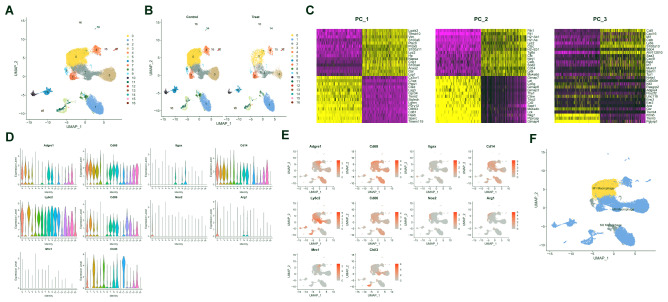
Cell clustering and annotation of scRNA-seq data. Note: **(A)** Visualization of the integrated results of UMAP clustering of two groups of macrophages, with cells divided into 17 subclusters (C0-C16), where different colors represent different subclusters. **(B)** Visualization of UMAP clustering results for macrophages in the control group and drug-treated group separately, with cells divided into 17 subclusters (C0-C16), where different colors represent different subclusters. **(C)** Top 3 PCA results and heatmap of gene expression for each cell sample, with purple indicating upregulation and yellow indicating downregulation. **(D)** Violin plots of the expression of macrophage marker genes in each cluster. **(E)** Violin plots of the expression of macrophage marker genes in each cluster. **(F)** Annotation of macrophage UMAP plots based on marker gene expression

To cluster the cells, we then applied the Louvain algorithm to iteratively group the cells together. This process was implemented using the FindCluster function, which includes a resolution parameter to control the granularity of the downstream clusters. Increasing the value would result in more subclusters. Finally, we divided all macrophages from the control and treatment groups into 17 different clusters (Fig. 1A). Among them, clusters 0, 2, 3, and 4 were more abundant in the control group, while cluster 1 was more abundant in the treatment group (Fig. 1B). Here, we present the top 3 results of the PCA analysis of each cell sample, as well as a heatmap showing the expression levels of the top 3 PCs (Fig. 1C).

Next, we labeled the macrophages with common marker genes such as Adgre1, Cd68, Itgax, Cd14, Ly6c2, Cd86, Nos2, Arg1, Mrc1 (Cd206), and Chil3. The results showed that the M1 macrophage markers Nos2 and Cd86 were highly expressed in cluster 0, while the M2 macrophage markers Arg1 and Cd206 were highly expressed in clusters 8 and 9 (Fig. 1D-E). Therefore, we manually annotated cluster 0 as M1 macrophages and clusters 8 and 9 as M2 macrophages, while the other clusters were annotated as M0 macrophages. The annotation results are shown in the visualization of the cell clusters (Fig. 1F).

### Effects of vancomycin-loaded calcium sulfate on macrophage polarization and gene expression in staphylococcus aureus infections

**Fig. 2:**
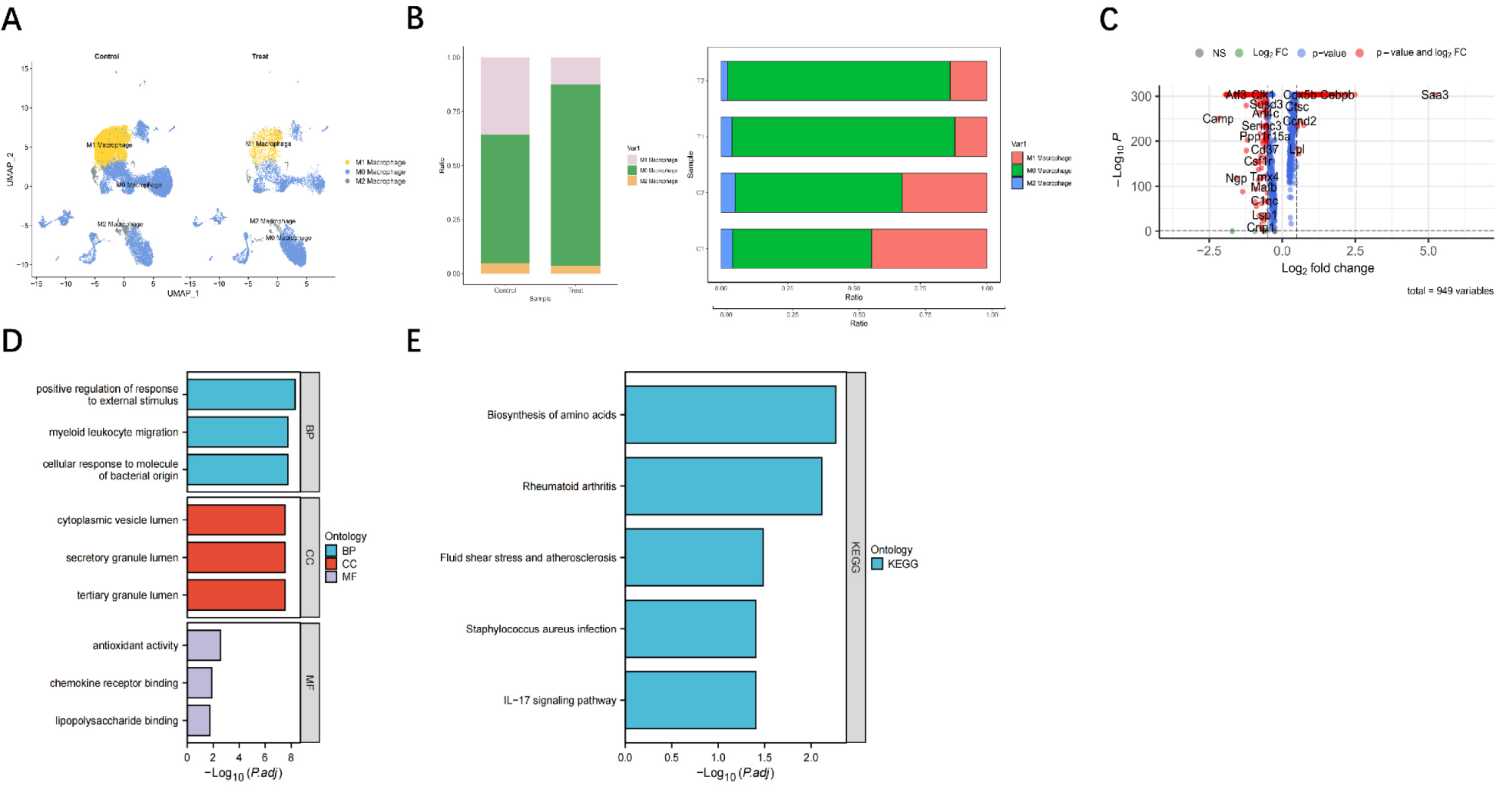
Differential gene analysis of macrophages before and after vancomycin treatment. Note: **(A)** Subgroup distribution of annotated cells in two samples. **(B)** Proportions of M0, M1, and M2 macrophages in the two samples. **(C)** Volcano plot showing differential gene expression in macrophages between the two samples. (D) Bar plot showing GO enrichment of differentially expressed genes, with red, blue, and yellow representing BP, CC, and MF, respectively. **(E)** Bar plot showing KEGG enrichment of differentially expressed genes

To further investigate the differences between the control and drug-treated macrophage groups, we generated UMAP plots of annotated cell clusters for both groups (Fig. 2A). The analysis of these plots revealed disparities in the proportions of M1 and M2 macrophages between the control and treatment groups, suggesting that drug treatment led to a distinct polarization of macrophages compared to the control. Subsequently, we conducted a comprehensive analysis of the divergent subtype distribution between the control and drug-treated macrophages. The results demonstrated a significant increase in the abundance of M1 macrophages in the control group compared to the treatment group (Fig. 2B), indicating that Vancomycin-Loaded Calcium Sulfate may promote M2 macrophage polarization. Next, we employed a volcano plot (Fig. 2C) to examine the differential gene expression profiles of macrophages in the control and treatment groups. Through careful screening, we identified 86 differentially expressed genes, including 48 significantly upregulated genes and 38 significantly downregulated genes. These changes in gene expression likely reflect the impact of the drug on macrophage polarization. Moreover, several genes related to macrophage polarization and immune responses exhibited increased expression in the Vancomycin-Loaded Calcium Sulfate-treated macrophages. These genes encompass a range of important cytokines and signaling molecules, which provide valuable insights into how Vancomycin-Loaded Calcium Sulfate regulates macrophage gene expression to combat Staphylococcus aureus infections.

### Differential gene expression and key regulators of macrophage polarization

To investigate the potential regulators of macrophage polarization, we performed GO and KEGG enrichment analyses using single-cell transcriptomic data. The results revealed that among the 86 differentially expressed genes, they were mainly enriched in biological processes, including “Positive regulation of response to external stimulus,” “Cellular response to molecule of bacterial origin,” and “Myeloid leukocyte migration.” In terms of cellular components, the differentially expressed genes were predominantly enriched in “Cytoplasmic vesicle lumen,” “Secretory granule lumen,” and “Tertiary granule lumen.” Concerning molecular functions, the differentially expressed genes showed significant enrichment in “Antioxidant activity,” “Chemokine receptor binding,” and “Lipopolysaccharide binding” (Fig. 2D). Additionally, the KEGG analysis indicated that the differentially expressed genes were associated with signaling pathways such as “Rheumatoid arthritis,” “Staphylococcus aureus infection,” and “IL − 17 signaling pathway” (Fig. 2E).

To identify key genes through which vancomycin-loaded calcium sulfate may affect macrophage polarization, we inputted those above 86 differentially expressed genes into the String website to obtain protein-protein interaction networks. MBD2 was found to occupy a central position in a gene network closely associated with macrophage polarization (Figure S3A-B). This suggests that MBD2 may directly or indirectly regulate other genes within the network. Furthermore, the KEGG enrichment analysis revealed MBD2’s involvement in the “Staphylococcus aureus infection” signaling pathway. Based on these findings, we speculate that MBD2 may play a crucial role in regulating macrophage polarization in the context of vancomycin-loaded calcium sulfate.

### Identification of key transcription factors driving macrophage differentiation in vancomycin-loaded calcium sulfate

**Fig. 3:**
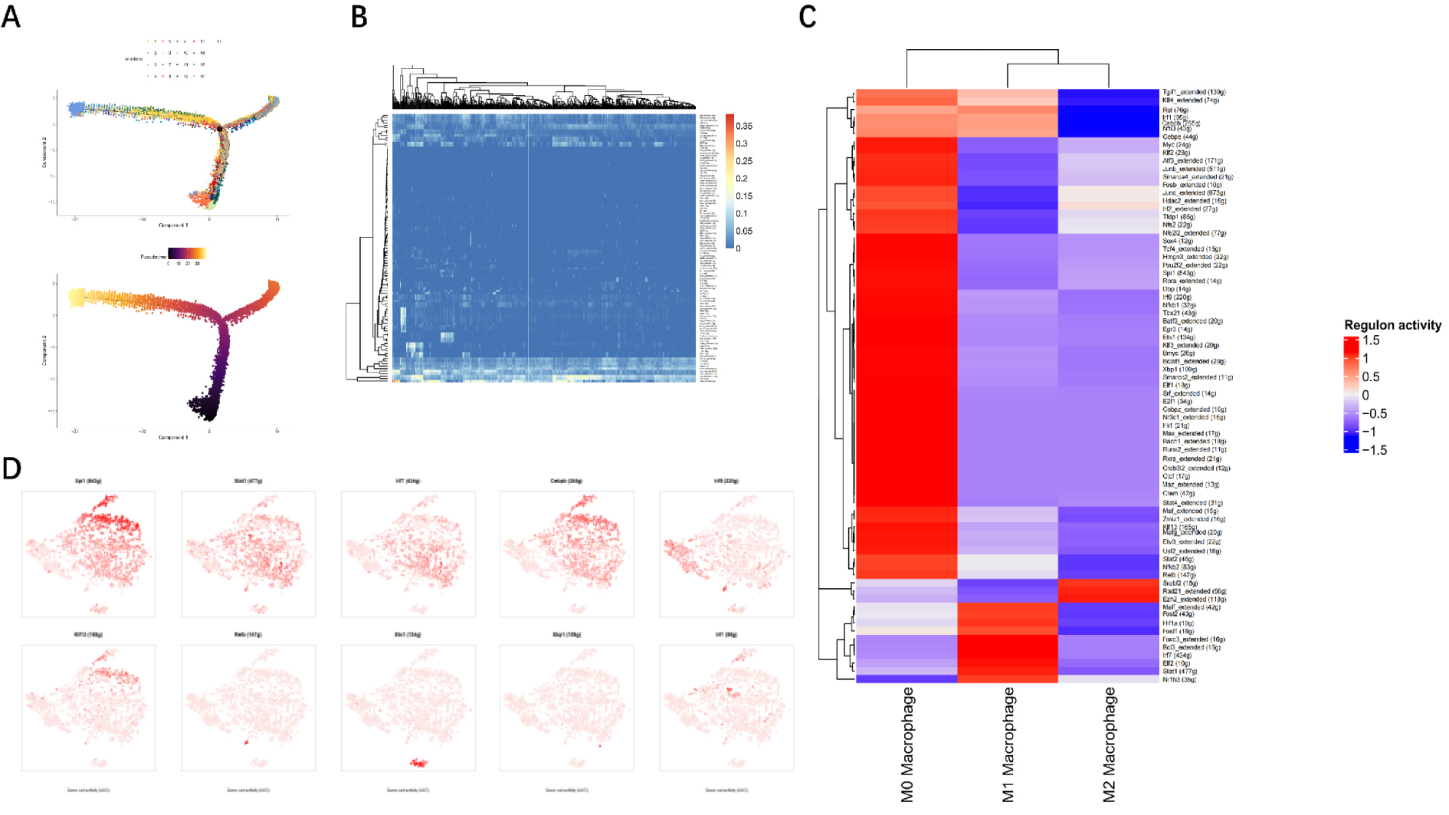
Development trajectory and transcription factor analysis of macrophages. Note: **(A)** Pseudotime analysis. **(B)** Heatmap showing the relative expression of transcription factor genes in each macrophage subcluster. **(C)** Heatmap showing the normalized activity of predicted transcription factors in each macrophage subcluster using pySCENIC. **(D)** Expression levels of highly active transcription factors in different cell subclusters of macrophage samples, with darker red indicating higher expression levels

We speculate that MBD2 may play a crucial role in regulating macrophage polarization in the process of Vancomycin-Loaded Calcium Sulfate. However, the process of MBD2 differentiation in macrophages remains unclear. Next, we performed a pseudo-temporal analysis of the data to infer the differentiation trajectory of macrophages (Fig. 3A). Macrophage differentiation is governed by a complex transcription factor network, whereby these transcription factors regulate each other’s functionality through interaction with their cofactors and downstream genes. Therefore, we evaluated the specific expression of TFs and regulatory networks in macrophages and their subtypes using SCENIC (Fig. 3B-C). We found that SPI1, STAT1, IRF7, CEBPB, IRF8, KLF13, RELB, ETS1, XBP1, and IRF1 exhibited the highest expression and activity levels in the regulatory network of macrophages, potentially representing the key TFs driving this differentiation pathway (Fig. 3D).

### Effects of vancomycin-loaded calcium sulfate on MBD2 expression and macrophage polarization

**Fig. 4:**
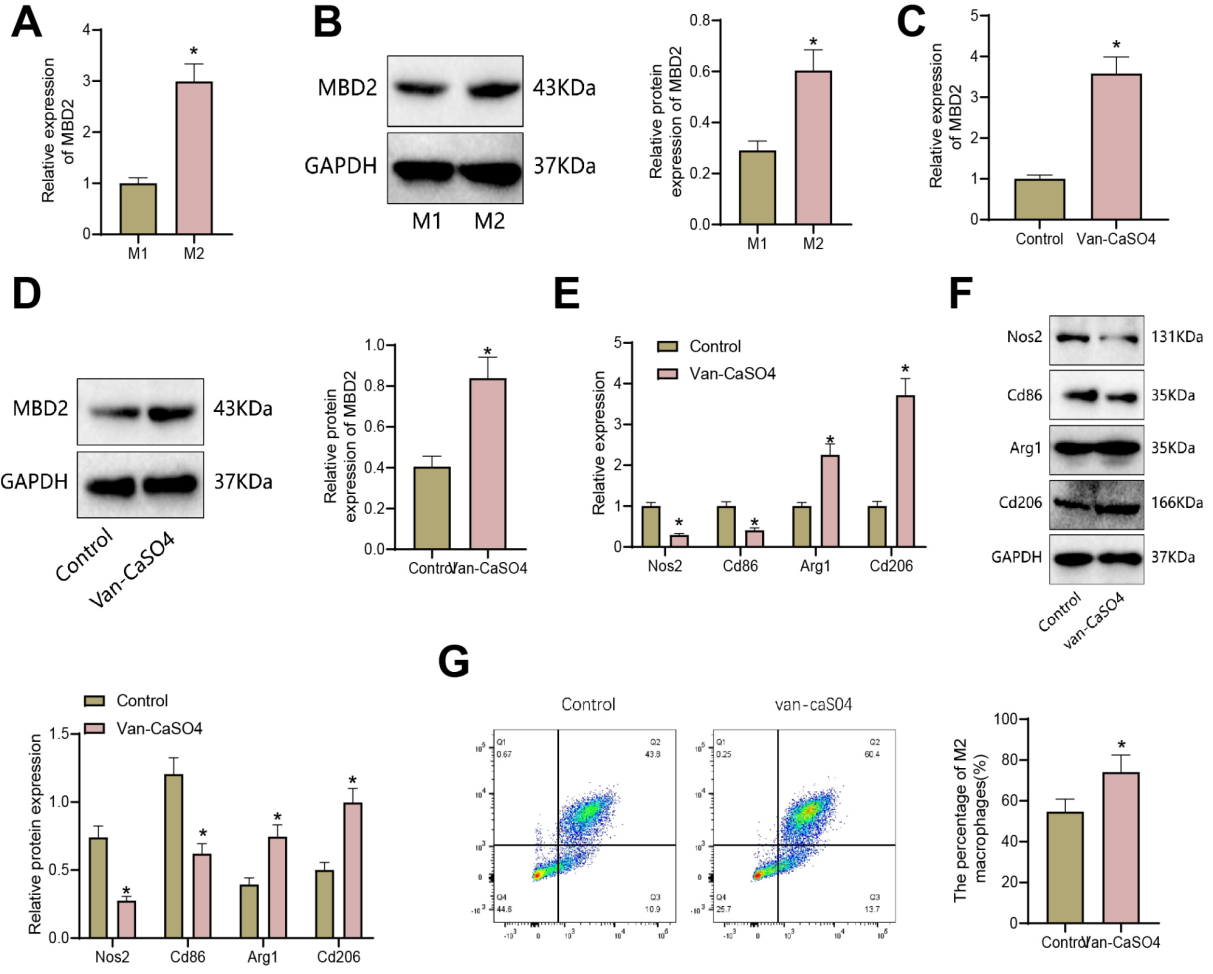
Influence of Vancomycin-Loaded Calcium Sulfate on M2 macrophage polarization through MBD2 expression. Note: **(A-B)** Expression levels of MBD2 in M1 polarized macrophages and M2 polarized macrophages detected by RT-qPCR and WB. **(C-D)** Expression changes of MBD2 in THP-1 cells after treatment with Vancomycin-Loaded Calcium Sulfate detected by RT-qPCR and WB. **(E-F)** Expression of M1 and M2 macrophage markers in THP-1 cells after treatment with Vancomycin-Loaded Calcium Sulfate detected by RT-qPCR and WB. **(G)** Proportion of M2 macrophages (CD206 + CD11b+) in THP-1 cells after treatment with Vancomycin-Loaded Calcium Sulfate detected by flow cytometry. * indicates *p* < 0.05 compared to the M1 or control group. Cell experiments were performed in triplicate

First, the THP-1 cell line was induced into M1 macrophages and M2 macrophages. RT-qPCR and Western blot were used to detect the expression levels of MBD2 in M1 polarized macrophages and M2 polarized macrophages. It was found that compared to M1 macrophages, MBD2 mRNA and protein levels were significantly increased in M2 macrophages (Fig. 4A-B).

To validate the effect of Vancomycin-Loaded Calcium Sulfate on MBD2 expression and polarization in macrophages, we first examined the expression changes of MBD2 in THP-1 cell line after treatment with Vancomycin-Loaded Calcium Sulfate using RT-qPCR and Western blot. The results showed that compared to the Control group, the mRNA and protein levels of MBD2 in THP-1 cells were significantly increased in the Van-CaSO4 group (Fig. 4C-D).

RT-qPCR and Western blot were used to detect the expression of M1 macrophage markers Nos2 and Cd86 and M2 macrophage markers Arg1 and Cd206 in the THP-1 cell line after treatment with Vancomycin-Loaded Calcium Sulfate. The results showed that compared to the Control group, the expression of M1 macrophage markers Nos2 and Cd86 was significantly reduced, while the expression of M2 macrophage markers Arg1 and Cd206 was significantly increased in the Van-CaSO4 group (Fig. 4E-F).

Flow cytometry detected the proportion of M2 macrophages (CD206 + CD11b+) in the THP-1 cell line after treatment with Vancomycin-Loaded Calcium Sulfate. The results showed that compared to the Control group, the proportion of M2 macrophages was significantly increased in the Van-CaSO4 group (Fig. 4G).

These results indicate that Vancomycin-Loaded Calcium Sulfate upregulates the expression of MBD2 in macrophages and promotes the polarization of M2 macrophages.

### MBD2 knockout inhibits M2 macrophage polarization and reverses the effect of vancomycin-loaded calcium sulfate

**Fig. 5:**
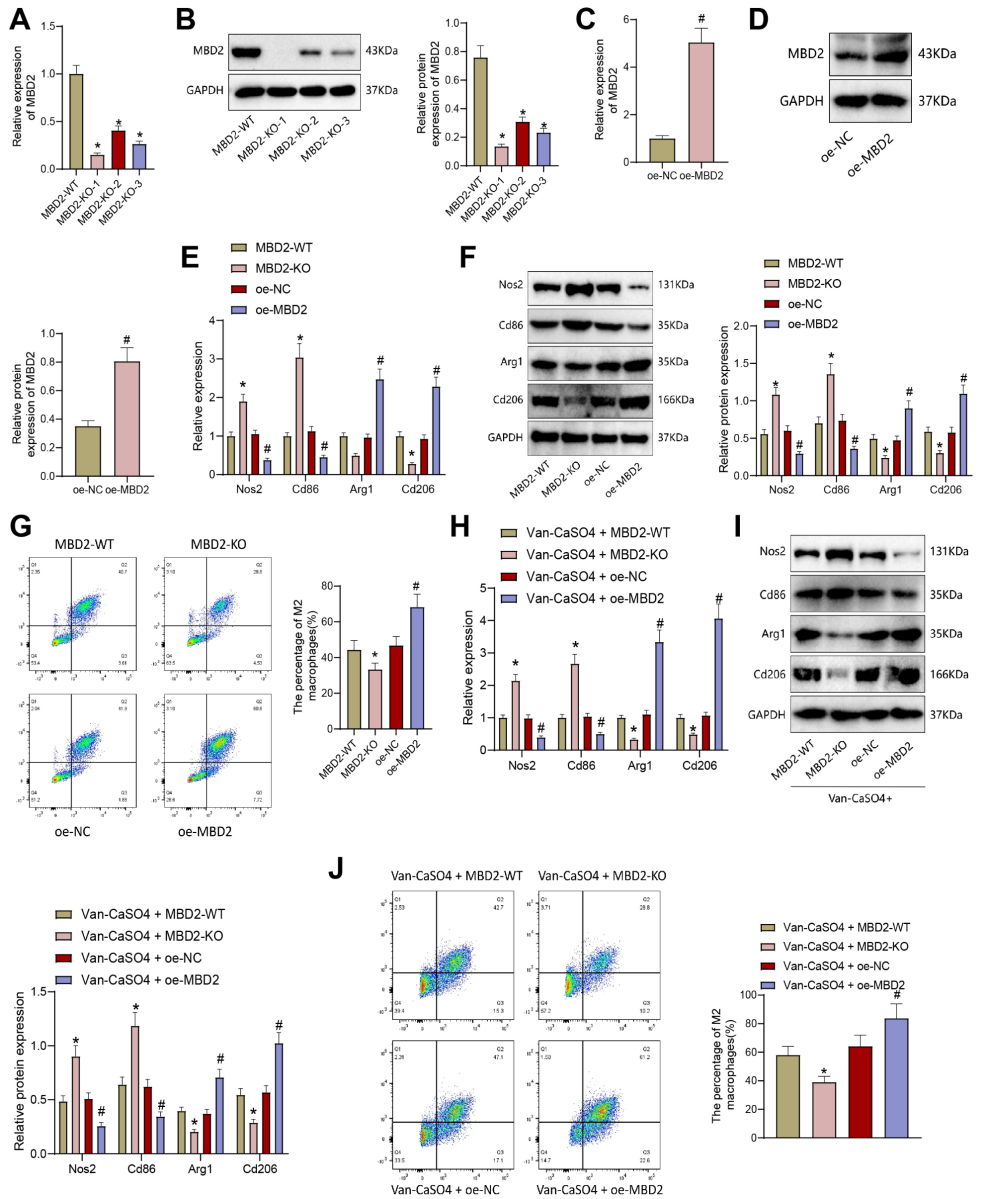
Effects of MBD2 knockout or overexpression on M2 macrophage polarization. Note: **(A)** Changes in MBD2 mRNA expression in MBD2-KO THP-1 cells constructed using CRISPR/Cas9 gene editing technology detected by RT-qPCR. **(B)** Changes in MBD2 mRNA expression in MBD2-KO THP-1 cells constructed using CRISPR/Cas9 gene editing technology detected by WB. **(C)** Changes in MBD2 mRNA expression in THP-1 cells after overexpression of MBD2 detected by RT-qPCR. **(D)** Changes in MBD2 mRNA expression in THP-1 cells after overexpression of MBD2 detected by WB. **(E-F)** Expression of M1 and M2 macrophage markers in THP-1 cells after overexpression or silencing of MBD2 detected by RT-qPCR and WB. **(G)** Proportion of M2 macrophages (CD206 + CD11b+) in THP-1 cells after overexpression or silencing of MBD2 detected by flow cytometry. **(H-I)** Expression of M1 and M2 macrophage markers in THP-1 cells treated with Vancomycin-Loaded Calcium Sulfate and overexpressing or silencing MBD2 detected by RT-qPCR and WB. **(J)** The proportion of M2 macrophages (CD206 + CD11b+) in THP-1 cells treated with Vancomycin-Loaded Calcium Sulfate and overexpressing or silencing MBD2 detected by flow cytometry. * indicates *p* < 0.05 compared to MBD2-WT group or Van-CaSO4 + MBD2-WT group, # indicates *p* < 0.05 compared to oe-NC group or Van-CaSO4 + oe-NC group. Cell experiments were performed in triplicate

To evaluate the influence of MBD2 on macrophages, we employed CRISPR/Cas9 gene editing technology to generate MBD2 knockout THP-1 cells (MBD2-WT served as the wild-type control for MBD2 knockout). The expression level of MBD2 in the monoclonal cells was assessed using RT-qPCR and Western blot, and monoclonal cells with complete knockout (referred to as MBD2-KO) were selected for subsequent expansion and cultivation (Fig. 5A-B). Additionally, we established an MBD2 overexpression THP-1 cell line using lentivirus construction. RT-qPCR and Western blot analysis demonstrated a significant increase in the mRNA and protein levels of MBD2 in the oe-MBD2 cells compared to the oe-NC group (Fig. 5C-D).

RT-qPCR and Western blot analyses were performed to determine the expression levels of M1 macrophage markers Nos2 and Cd86 and M2 macrophage markers Arg1 and Cd206 in THP-1 cells following MBD2 knockout or overexpression. Compared to the MBD2-WT group, the MBD2-KO group showed significantly increased expression of Nos2 and Cd86, indicative of M1 macrophage polarization, and significantly decreased expression of Arg1 and Cd206, indicative of M2 macrophage polarization. In contrast, the oe-MBD2 group exhibited significantly reduced expression of Nos2 and Cd86 and significantly increased expression of Arg1 and Cd206 compared to the oe-NC group (Fig. 5E-F).

Flow cytometry analysis was employed to assess the proportion of M2 macrophages (CD206 + CD11b+) in THP-1 cells following MBD2 knockout or overexpression. The MBD2-KO group displayed a significant reduction in the proportion of M2 macrophages compared to the MBD2-WT group, while the oe-MBD2 group showed a significant increase in the proportion of M2 macrophages compared to the oe-NC group (Fig. 5G).

Subsequently, RT-qPCR and Western blot analyses were performed to assess the expression levels of Nos2 and Cd86 as M1 macrophage markers and Arg1 and Cd206 as M2 macrophage markers in THP-1 cells treated with Vancomycin-Loaded Calcium Sulfate. The Van-CaSO4 + MBD2-KO group exhibited a significant increase in Nos2 and Cd86 expression and a significant decrease in Arg1 and Cd206 expression compared to the Van-CaSO4 + MBD2-WT group. In contrast, the Van-CaSO4 + oe-MBD2 group displayed a significant decrease in Nos2 and Cd86 expression and a significant increase in Arg1 and Cd206 expression, compared to the Van-CaSO4 + oe-NC group (Fig. 5H-I).

Flow cytometry analysis was then conducted to determine the proportion of M2 macrophages (CD206 + CD11b+) in THP-1 cells treated with Vancomycin-Loaded Calcium Sulfate following MBD2 knockout or overexpression. The Van-CaSO4 + MBD2-KO group exhibited a significant reduction in the proportion of M2 macrophages compared to the Van-CaSO4 + MBD2-WT group, while the Van-CaSO4 + oe-MBD2 group showed a significant increase in the proportion of M2 macrophages compared to the Van-CaSO4 + oe-NC group (Fig. 5J).

Taken together, these results suggest that MBD2 knockout inhibits M2 polarization in macrophages and reverses the promoting effect of Vancomycin-Loaded Calcium Sulfate on M2 macrophage polarization.

### Promotion of M2 macrophage polarization and synergistic effect of vancomycin-loaded calcium sulfate and negative pressure wound therapy in the treatment of open fracture infection

**Fig. 6:**
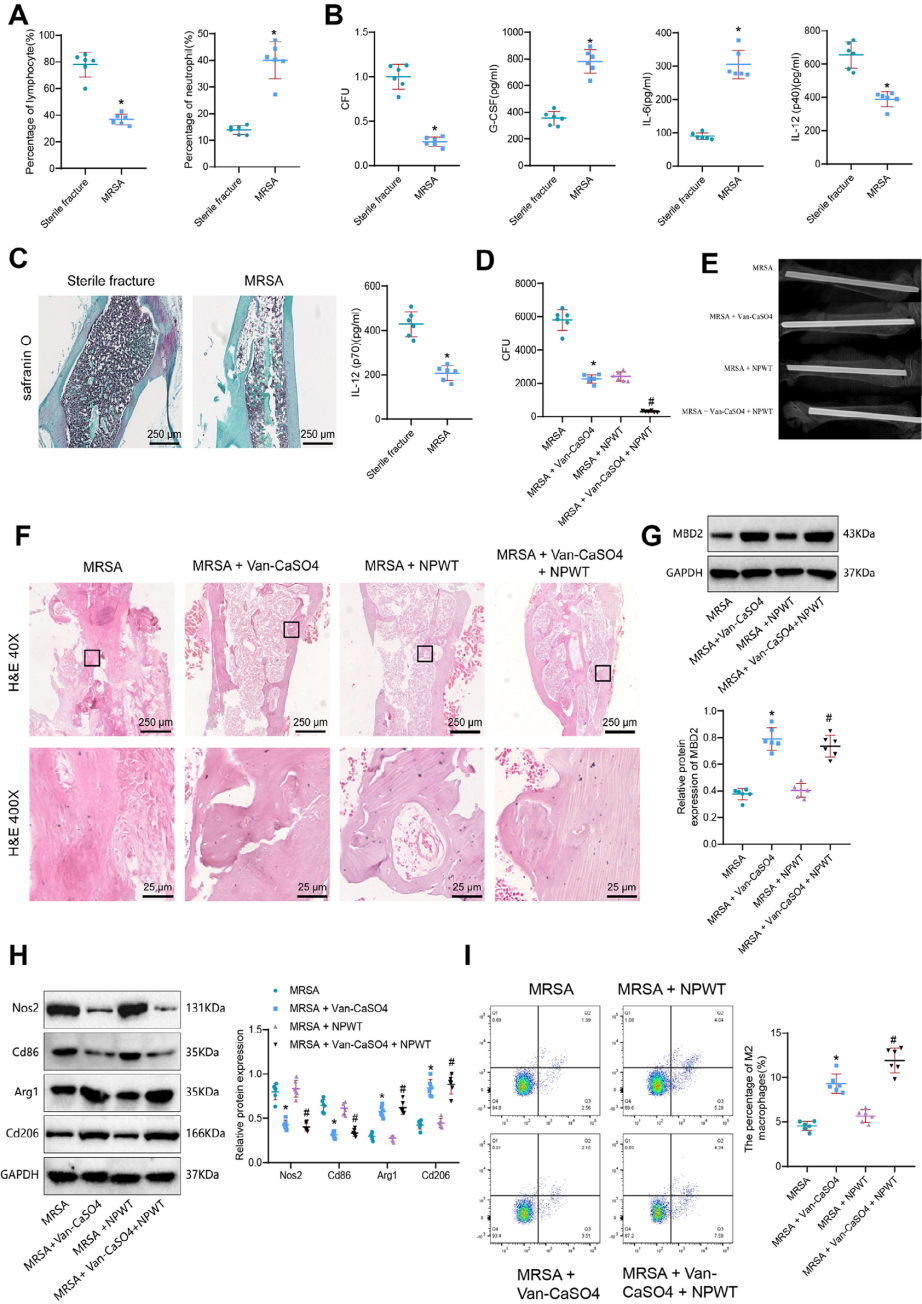
Combined treatment of Vancomycin-Loaded Calcium Sulfate and negative pressure drainage apparatus for combating open fracture infection. Note: **(A)** Percentage of lymphocytes and neutrophils in mouse blood on the 5th day of MRSA infection analyzed by CBC blood routine analysis. **(B)** Levels of G-CSF, IL-6, IL-12 (p40), and IL-12 (p70) in mouse serum detected by ELISA. **(C)** Safranin O staining of cartilage formation in mouse groups (Scar Bar = 1000 μm). **(D)** Bacterial CFU in mouse joint tissue detected by bacterial culture. **(E)** X-ray detection of skeletal repair in mouse joints. **(F)** H&E staining of tissue damage in mouse fractures (Scar Bar = 1000 μm). **(G)** Expression of MBD2 protein in mouse fracture tissues detected by WB. **(H)** Expression of M1 and M2 macrophage markers in mouse fracture tissues detected by WB. **(I)** Proportion of M2 macrophages (CD206 + CD11b+) in mouse fracture tissues detected by flow cytometry. * indicates *p* < 0.05 compared to the Sterile fracture or MRSA group. *n* = 6

To validate our findings, we established a mouse model of infected fracture. CBC blood analysis revealed that on day 5 of MRSA infection, mice in the MRSA group exhibited a significant decrease in lymphocyte percentage and a significant increase in neutrophil percentage compared to the Sterile fracture group (Fig. 6A). Additionally, a comparison of serum cytokine analysis showed that the expression of G-CSF and IL-6 was significantly increased in the MRSA infection samples compared to the Sterile fracture group, while the expression of IL-12 (p40) and IL-12 (p70) was decreased (Fig. 6B). Safranin O staining demonstrated that in the MRSA infection group, two weeks after infection, there was a decrease in proliferation and differentiation of granulation tissue, as well as a reduction in the area of cartilage callus compared to the Sterile fracture group (Fig. 6C). These results confirm the successful establishment of a mouse model of fracture infection.

Negative pressure wound therapy (NPWT) is an innovative medical device that promotes wound healing by creating negative pressure at the wound site [12, 29]. However, does NPWT interact with Vancomycin-Loaded Calcium Sulfate and influence macrophage polarization, thus participating in the resistance to open fracture infection? To test this hypothesis, we treated the mice in the infected fracture model with either Vancomycin-Loaded Calcium Sulfate alone, NPWT alone, or a combination of both.

The results of joint tissue bacterial culture indicated that, compared to the mice in the MRSA group, the CFU values of mice in all treatment groups showed a significant decrease in synovial tissue of the joints. Furthermore, the CFU values were lower in the mice treated with MRSA + Van-CaSO4 + NPWT compared to those treated with MRSA + Van-CaSO4 and MRSA + NPWT (Fig. 6D).

X-ray examination results showed that, compared to the mice in the MRSA group, the mice in all treatment groups exhibited a significant improvement in the degree of joint bone repair. Moreover, the mice treated with MRSA + Van-CaSO4 + NPWT showed a better degree of joint bone repair compared to those treated with MRSA + Van-CaSO4 and MRSA + NPWT (Fig. 6E).

H&E staining revealed that in the fracture tissues of mice infected with MRSA, there was a dense infiltration of lymphocytes, inadequate cartilage formation, disrupted cartilaginous callus area, and a lack of cartilaginous bridging. Conversely, the fracture tissues of mice treated with MRSA + Van-CaSO4, MRSA + NPWT, and MRSA + Van-CaSO4 + NPWT exhibited cartilaginous bridging and residual cartilage matrix, with a clear boundary of the healing tissue area and relatively organized tissue. Notably, the mice treated with MRSA + Van-CaSO4 + NPWT showed a significant presence of cartilage tissue and a clear boundary of the healing tissue area with well-organized tissue (Fig. 6F).

Western blot analysis of MBD2 protein expression in the fracture tissues of mice demonstrated that, compared to the mice in the MRSA group, the mice in the MRSA + Van-CaSO4 and MRSA + Van-CaSO4 + NPWT groups exhibited a significant increase in MBD2 protein expression in the synovial bone tissue (Fig. 6G).

Western blot analysis of the expression of M1 macrophage marker proteins Nos2 and Cd86, as well as M2 macrophage marker proteins Arg1 and Cd206, in the fracture tissues of mice revealed that, compared to the mice in the MRSA group, the THP-1 cell line from the mice in the MRSA + Van-CaSO4 and MRSA + Van-CaSO4 + NPWT groups showed a significant decrease in the expression of the M1 macrophage markers Nos2 and Cd86, and a significant increase in the expression of the M2 macrophage markers Arg1 and Cd206 (Fig. 6H).

Flow cytometry analysis revealed that, compared to the mice in the MRSA group, the proportion of M2 macrophages (CD206 + CD11b+) in the fracture tissues of mice in the MRSA + Van-CaSO4 and MRSA + Van-CaSO4 + NPWT groups was significantly increased (Fig. 6I).

Taken together, these results indicate that Vancomycin-Loaded Calcium Sulfate promotes M2 cell polarization through upregulation of MBD2 expression, thereby preventing open fracture infection in a mouse model. Moreover, the combined treatment of Vancomycin-Loaded Calcium Sulfate and NPWT exhibits a synergistic effect in combating open fracture infection.

## Discussion

This study primarily discovered that the Vancomycin-Loaded Calcium Sulfate combined with a negative pressure drainage device may prevent open fracture infection by modulating the macrophage polarization through the methyl-CpG binding protein MBD2. This finding significantly expands our understanding of anti-infective treatment strategies and provides a new therapeutic target [30]. Previous studies have extensively explored the role of macrophages in fracture infection and the function of MBD2 in regulating cellular function, but many aspects of the specific molecular mechanisms remain unclear [31–33]. The discovery in this study not only reveals the importance of MBD2 in macrophage polarization but also demonstrates the impact of Vancomycin-Loaded Calcium Sulfate on macrophages, providing a theoretical basis for further development and improvement of treatment strategies [11]. Furthermore, our study revealed that the combined application of VLCS and NPWT significantly enhances the M2 polarization of macrophages and boosts infection resistance. This finding suggests that VLCS and NPWT not only possess individual anti-infective properties but also exhibit synergistic effects when used together, thereby further enhancing treatment efficacy.

In the single-cell transcriptome sequencing part, we observed significant differences in gene expression patterns between the treatment and control groups of macrophages [34]. This result is consistent with previous findings on macrophage gene expression changes, but we further discovered that the expression levels of some polarization-related genes and infection response-related genes in the treatment group were significantly increased [35]. This finding fills the gap in previous research and reveals that Vancomycin-Loaded Calcium Sulfate may alter macrophage function and prevent fracture infection by regulating the expression of these key genes [36]. In the bioinformatics analysis, we found that MBD2 occupies a central position in the relevant gene network. Previous studies mainly focused on the role of MBD2 in DNA methylation, gene silencing, and other processes, while its role in macrophage polarization has yet to receive widespread attention [37]. This study first discovered the potential central regulatory role of MBD2 in macrophage polarization, providing a new perspective to understand the comprehensive function and regulatory mechanisms of MBD2 [11].

In the in vitro cell experiments and gene editing experiments, we found that the expression level of MBD2 can influence the impact of Vancomycin-Loaded Calcium Sulfate on macrophages. Previous studies have also reported the use of gene editing techniques to study the function of MBD2, but most of them focused on its role in tumor cells [38]. Our study is the first to discover that in macrophages, the expression of MBD2 directly affects the effect of Vancomycin-Loaded Calcium Sulfate, where MBD2 knockout weakens the response of macrophages to the drug, while overexpression enhances the response. In the in vivo experiments, we found a significant reduction in infection rate in the mouse fracture infection model treated with Vancomycin-Loaded Calcium Sulfate and negative pressure drainage device. This finding strengthens the evidence of the anti-infective effect of Vancomycin-Loaded Calcium Sulfate from previous studies and further reveals its potential mechanism of action [39]. Additionally, our study shows through X-ray imaging results that the treatment group has better bone repair than the control group, which has not been extensively reported in previous studies. Histological analysis of femur samples collected shows good recovery of bone tissue structure in the treatment group with a significant reduction in infection lesions. Further analysis revealed that the combined use of VLCS and NPWT significantly enhances M2 polarization of macrophages and improves infection resistance. This discovery indicates that not only do VLCS and NPWT individually possess anti-infective properties, but their combined utilization can generate a synergistic effect, thereby further enhancing therapeutic efficacy. In previous studies, there has been insufficient research on the changes in bone tissue after infection and their relationship with treatment outcomes [40]. Our study provides comprehensive histological evidence for the first time, illustrating that the therapeutic effects of Vancomycin-Loaded Calcium Sulfate are not limited to infection control but are also beneficial for bone tissue repair [41].

## Conclusion

**Fig. 7:**
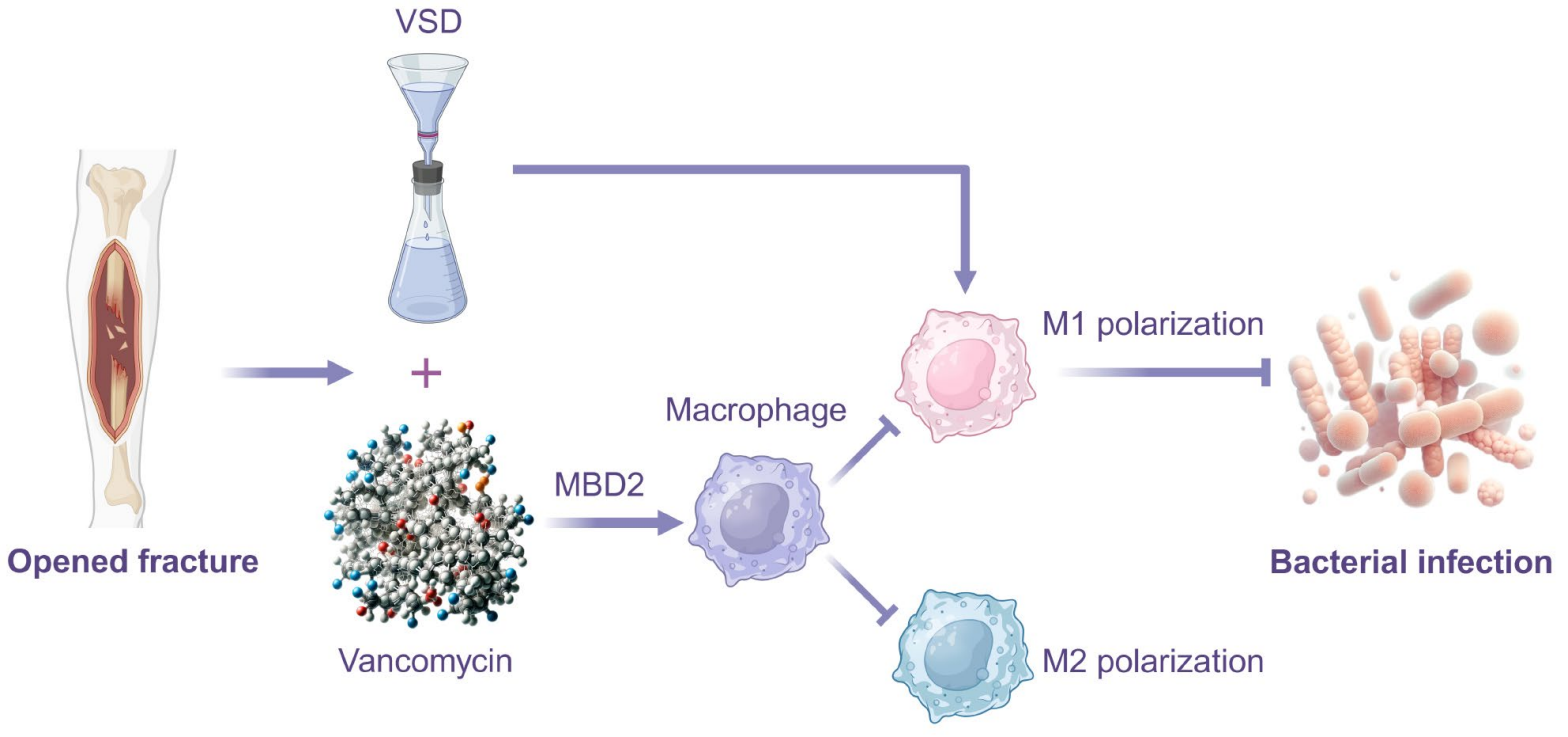
Mechanism diagram of Vancomycin-Loaded Calcium Sulfate-mediated macrophage polarization through MBD2 regulation in preventing open fracture infection

In summary, our study discovered that Vancomycin-Loaded Calcium Sulfate can prevent open fracture infection by regulating MBD2-mediated macrophage polarization (Fig. 7). This finding not only enhances our understanding of fracture infection treatment strategies but also provides new therapeutic targets, offering new directions for future drug development. This finding not only enhances our understanding of fracture infection treatment strategies but also provides new therapeutic targets, offering new directions for future drug development. We look forward to applying this discovery to clinical practice to better assist patients with fracture infections.

Despite the significant findings of our research, there are limitations that should be acknowledged. For example, our experimental model primarily relied on mice, and the physiological mechanisms of humans and mice are not entirely the same, thereby requiring further clinical research to validate our findings. Additionally, the specific regulatory mechanisms of MBD2 in macrophages need further exploration. Future studies could attempt to combine structural biology, proteomics, and other multi-level approaches to comprehensively reveal the role of MBD2 in macrophage polarization.

### Electronic supplementary material

Below is the link to the electronic supplementary material.

Supplementary Material 1. Figure S1. Bioinformatics workflow.

Supplementary Material 2. Figure S2. Cell Clustering and Annotation of scRNA-seq Data. Note: (A) Visualization of QC metrics: the left panel represents the number of mRNA species, the middle panel represents the number of captured mRNAs, and the right panel represents the proportion of mitochondrial RNA. Cells were filtered based on these criteria, filtering out cells with more than 2,000 or fewer than 200 mRNA species, and cells with more than 5% mitochondrial RNA. (B) Scatter plot showing the relationship between the number of mitochondria and the number of genes, and the relationship between the number of cells and the number of genes. (C) Highly variable genes were screened using variance analysis, with red representing the top 2000 highly variable genes and black representing low variability genes. (D) PCA analysis of cell distribution on PC_1 and PC_2, where each point represents a cell. (E) Distribution of standard deviation of PCs, with significant PCs having a larger standard deviation.

Supplementary Material 3. Figure S3. Co-expression network analysis results. Note: (A) Protein interaction network encoded by differentially expressed genes (MBD2 labeled in the network). (B) Bar chart of hub genes in the protein interaction network.

## Data Availability

No datasets were generated or analysed during the current study.
