# Supplementary figures and images for: Novel insights into vancomycin-loaded calcium sulfate and negative pressure wound therapy in preventing infections in open fractures
Source: J Orthop Surg Res. 2024 Aug 29;19:517. doi: 10.1186/s13018-024-04931-5 (PMC11360527; doi:10.1186/s13018-024-04931-5)

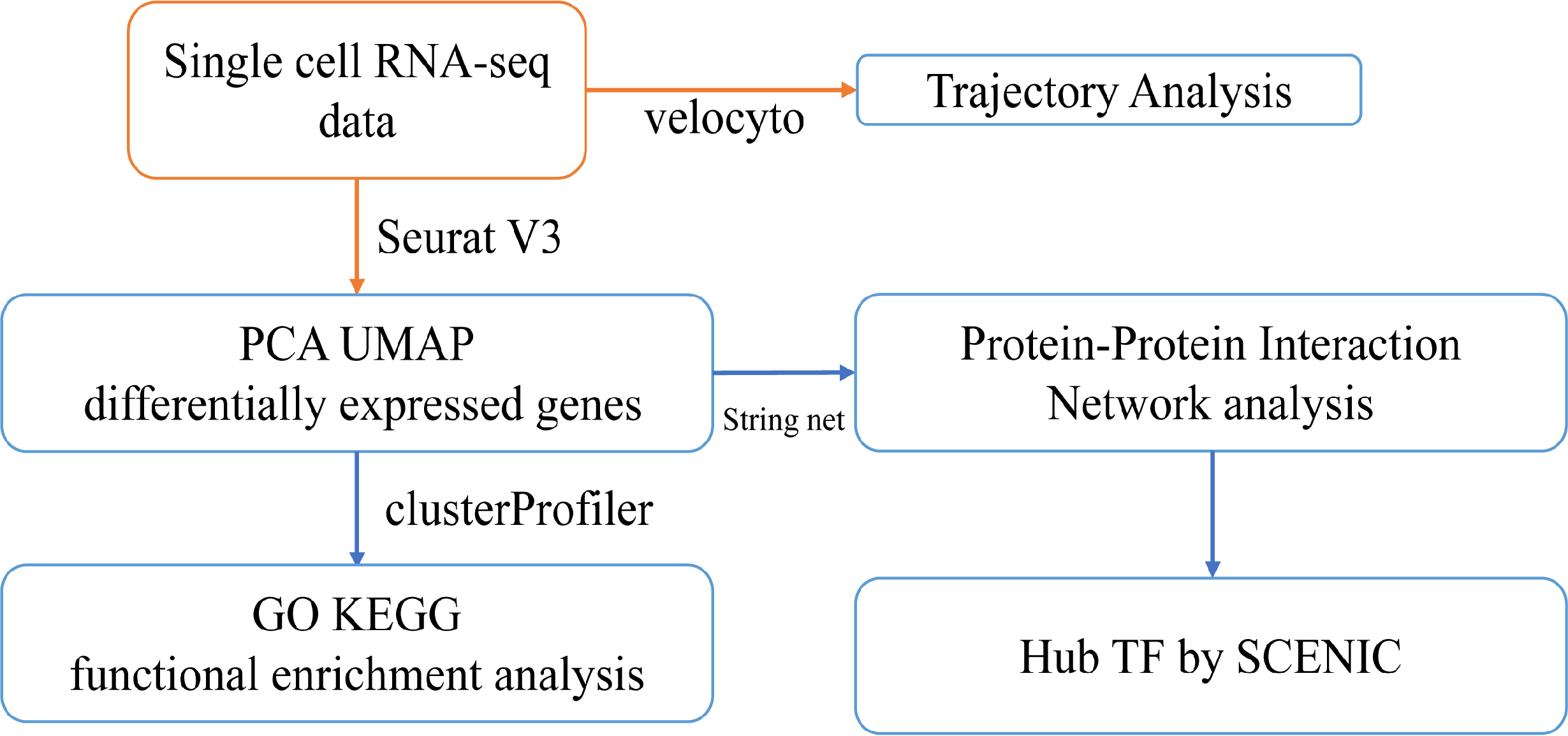

Supplement: Supplementary file 1 — Supplementary Material 1. Figure S1. Bioinformatics workflow. [file 13018_2024_4931_MOESM1_ESM.jpg]

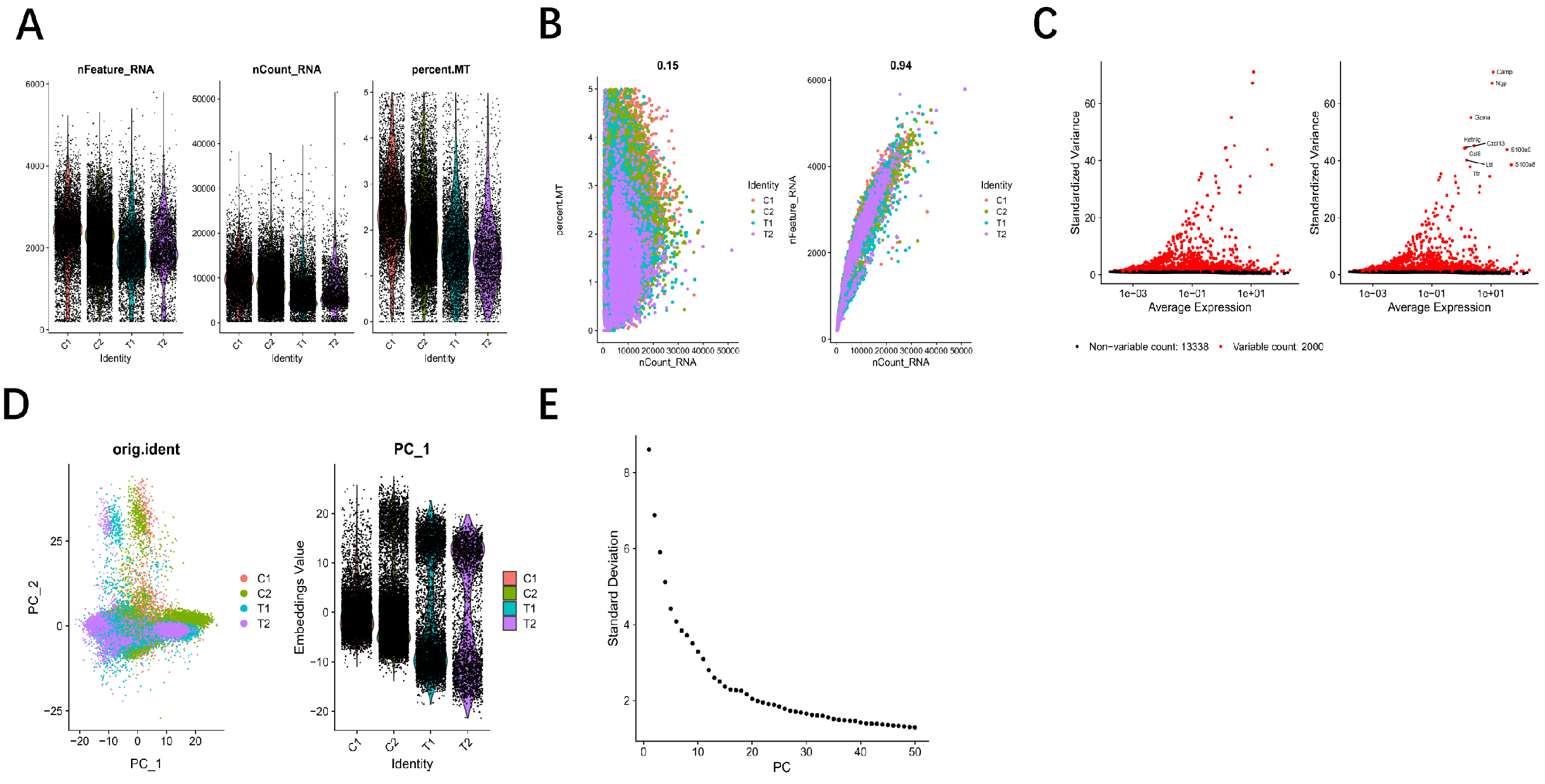

Supplement: Supplementary file 2 — Supplementary Material 2. Figure S2. Cell Clustering and Annotation of scRNA-seq Data. Note: (A) Visualization of QC metrics: the left panel represents the number of mRNA species, the middle panel represents the number of captured mRNAs, and the right panel represents the proportion of mitochondrial RNA. Cells were filtered based on these criteria, filtering out cells with more than 2,000 or fewer than 200 mRNA species, and cells with more than 5% mitochondrial RNA. (B) Scatter plot showing the relationship between the number of mitochondria and the number of genes, and the relationship between the number of cells and the number of genes. (C) Highly variable genes were screened using variance analysis, with red representing the top 2000 highly variable genes and black representing low variability genes. (D) PCA analysis of cell distribution on PC_1 and PC_2, where each point represents a cell. (E) Distribution of standard deviation of PCs, with significant PCs having a larger standard deviation. [file 13018_2024_4931_MOESM2_ESM.jpg]

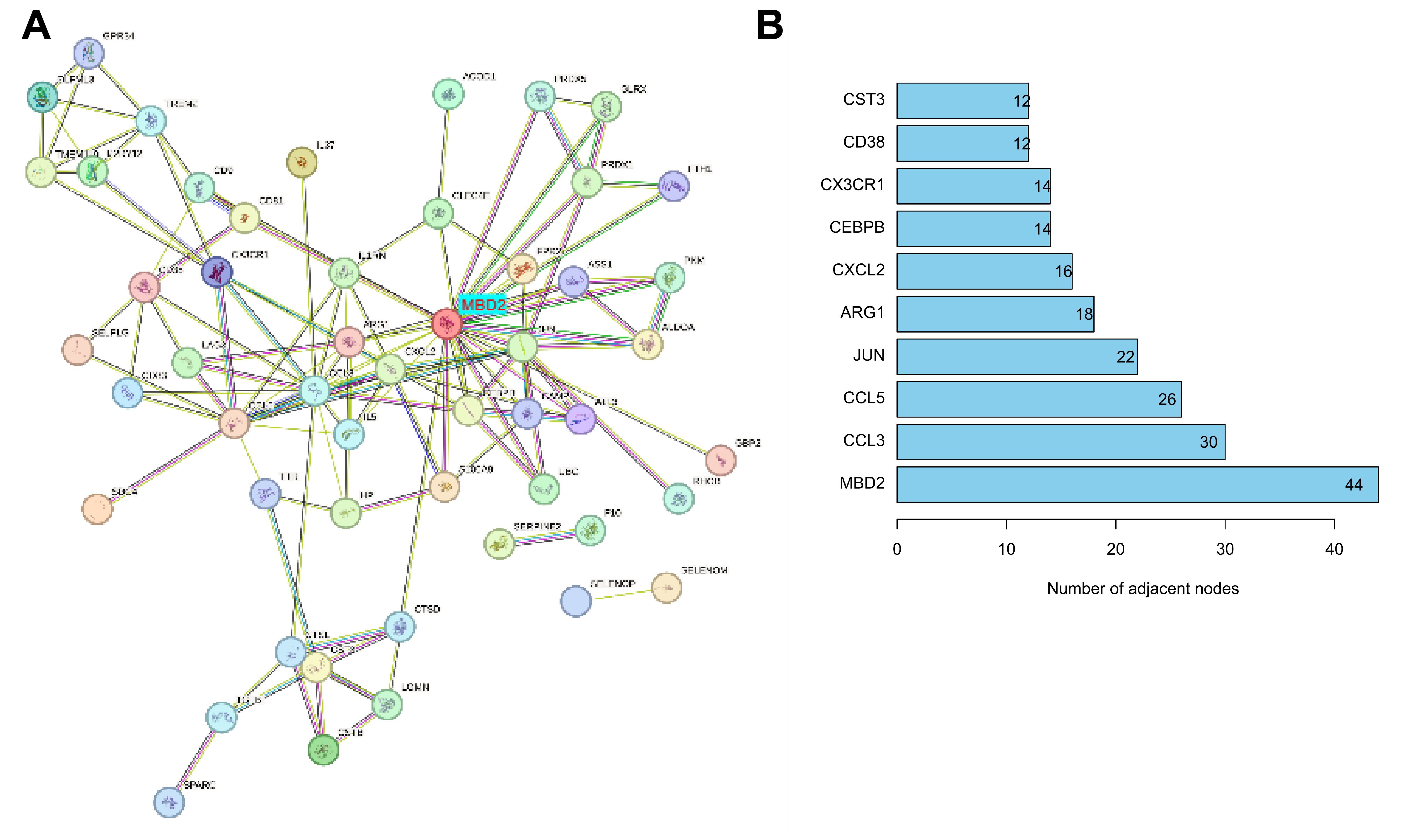

Supplement: Supplementary file 3 — Supplementary Material 3. Figure S3. Co-expression network analysis results. Note: (A) Protein interaction network encoded by differentially expressed genes (MBD2 labeled in the network). (B) Bar chart of hub genes in the protein interaction network. [file 13018_2024_4931_MOESM3_ESM.jpg]
